# The Behavioral Response of Larval Amphibians (Ranidae) to Threats from Predators and Parasites

**DOI:** 10.1371/journal.pone.0049592

**Published:** 2012-11-20

**Authors:** Dorina Szuroczki, Jean M. L. Richardson

**Affiliations:** Department of Biological Sciences, Brock University, St. Catharines, Ontario, Canada; California State University Fullerton, United States of America

## Abstract

Organisms are exposed to strong selective pressures from several sources, including predators and pathogens. Response to such interacting selective pressures may vary among species that differ in life history and ecology in predictable ways. We consider the impact of multiple enemies (fish predators and trematode parasites) on the behavior of larvae of three anuran species (*Lithobates* ( = *Rana*) *sylvaticus*, *L. clamitans* and *L. catesbeianus*). We show that the three ranid species differ in response to the trade-off imposed by the simultaneous presence of fish predators and trematode parasites in the environment. Two more permanent pond breeders (*L. clamitans* and *L. catesbeianus*), which commonly encounter parasites and fish, increased activity when in the combined presence of parasites and a fish predator, resulting in a relatively lower parasite encystment rate. In contrast, the temporary pond breeder (*L. sylvaticus*), which does not commonly encounter fish in the wild, decreased activity in the combined presence of a fish predator and parasites similar to when only the predator was present. For *L. sylvaticus,* this suggests that the presence of an unknown predator poses a greater threat than parasites. Further, the presence of fish along with parasites increased the susceptibility of both *L. sylvaticus* and *L. clamitans* to trematode infection, whereas parasite infection in *L. catesbeianus* was unaffected by the presence of fish. Unpalatability to fish may allow some species to respond more freely to attacking parasites in the presence of fish. The results from this study highlight the importance of considering multiple selective pressures faced by organisms and how this shapes their behavior.

## Introduction

Animals are confronted with trade-offs on a daily basis. However, none is as fundamental as the trade-off between the need to eat while simultaneously evading predators [1]. For example, almost all free-living animals must be active in order to acquire resources and obtain mates, but this activity also makes prey vulnerable to predators [2]. This is particularly clear within the larval anuran system. Larval anurans are filter feeders, sometimes detaching algae and detritus from the substrate that can then be filtered out of the water column [3]. In general, increased activity is associated with increased food intake and faster growth rates [4]. A trade-off occurs because a more active tadpole is more conspicuous to predators and therefore more vulnerable relative to an inactive counterpart [4]. Reduced activity in the presence of predators has been empirically demonstrated in larvae of several anuran species, e.g. [5], [6].

Larval anurans have other defense mechanisms as protection from predators. For example, some anuran larvae have noxious or toxic skin secretions [7]. Others exhibit morphological phenotypic plasticity, altering their shape (e.g. tail fin depth) in response to predators, e.g. [8]. Changes in morphology can thus minimize encounter rates or decrease the likelihood that the predator will successfully capture the tadpole upon encounter [9].

While the importance of predators in aquatic systems has been well-studied, less well-studied are the parasites commonly present in the same aquatic systems, even though parasite effects can be similar to that of predators [10]. Echinostomes are a group of trematode parasites now known to be an important disease agent in amphibian populations [11]. Echinostomes have a complex life cycle, requiring three hosts. *Echinostoma trivolvis* uses a snail (*Planorbella trivolvis*) as the first intermediate host; free-swimming cercariae emerge from the snail and can then infect a wide range of secondary intermediate hosts, including tadpoles [12]. Once cercariae contact a tadpole, the cercariae crawl much like an inchworm along the epidermis, entering the cloaca and encysting in the developing kidneys [13]–[15]. In response to the presence of parasites, tadpoles increase activity [16], [17] and exhibit numerous unique behaviors (e.g. explosive swimming with high angular accelerations) [18]. Increased activity decreases subsequent infection prevalence in tadpoles [19].

**Figure 1:**
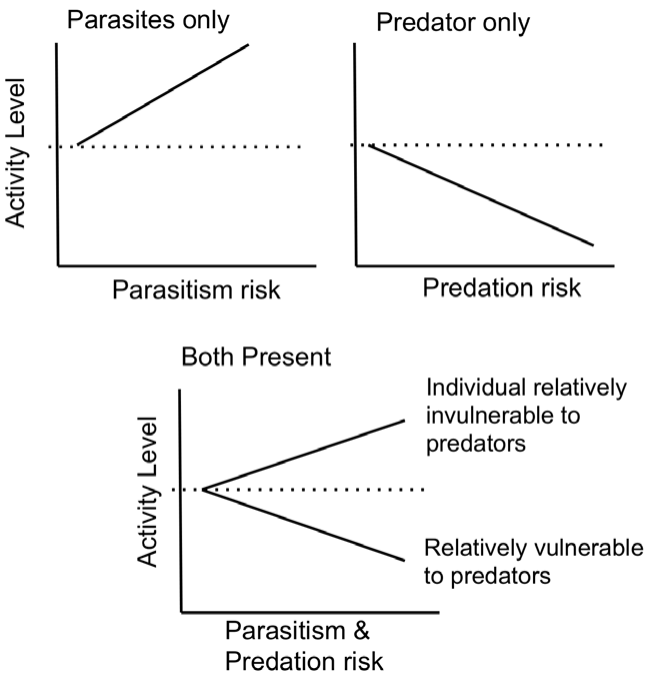
Response of tadpoles to the presence of parasites and predators and the hypothesized response to the presence of both, which will depend on the relative vulnerability of the tadpole to the predator. The dotted line in each plot represents baseline activity level, i.e. the activity level of the tadpole when no predator or parasite is present. We hypothesize that species such as *Lithobates sylvaticus*, which is highly vulnerable to fish predators, will show decreased activity in the presence of both fish and parasites, while species such as *L. catesbeianus*, which is relatively invulnerable to fish predators will increase activity in the presence of both fish and parasites.

If an increase in activity is used to lower parasitism rates, this is directly counter to the optimal response when a fish predator is present: reduction of activity in the presence of a predator will reduce the tadpole's ability to shake off not-yet-attached cercariae on their skin. Thus, a trade-off, or conflict, occurs between antipredator and antiparasite behavior [20]. This second trade-off (recall that there is also a trade-off between growth rate and predator avoidance mediated through activity) generates an additional component that must be considered for a mechanistic understanding of larval anuran behavior. Since predation renders individual fitness zero, we might expect that predator avoidance will outweigh avoidance of parasites in determining behavior. However, the cost of echinostome infection can be high in terms of reduced growth and survivorship, and infection is fatal at early tadpole stages [11], [21]. Also, as noted above, predation risk may vary among larval anurans depending on the presence of other anti-predator traits. In a previous study, we demonstrated that three sympatric *Lithobates* species vary in palatability to fish (*Lepomis*) predators [22]. *Lithobates catesbeianus*, found frequently in large lakes with fish as top predators, have limited vulnerability to fish predators because fish appear to find them unpalatable and will avoid them once experienced. *Lithobates sylvaticus* tadpoles, on the other hand, were always readily consumed by fish. Finally, while experienced fish consumed fewer *L. clamitans*, fish predation was reduced to a much smaller degree than for *L. catesbeianus*. We predict therefore that relative to baseline activity levels, in the presence of both *Echinostoma* and *Lepomis* visual and chemical cues, *L. catesbeianus* will behave similarly to when in the presence of parasites alone (i.e. have increased activity), while *L. sylvaticus* and *L. clamitans* behavior will be more similar to their response when in the presence of the fish predator alone (i.e. have decreased activity) (Fig. 1). We test this prediction, using behavioral responses of larvae from these three anuran species, *L. sylvaticus*, *L. clamitans*, and *L. catesbeianus*, to the combined presence of parasites and fish. In addition, we quantify parasitism prevalence and intensity in tadpoles of each species under each treatment combination that included parasites to assess the relationship between behavioral response and parasitism.

## Methods

### Animal Collection and Husbandry

All tadpoles were collected from Algonquin Park, Ontario, Canada (45°35′N, 78°31′W) in 2008. Nine *L. sylvaticus* egg masses were collected from Bat Lake at the Wildlife Research Station and housed in glass bowls at 5°C prior to hatching (necessary for hatching success in this species). While *L. sylvaticus* females attach egg masses to vegetation ∼10 cm below the water surface, with many females laying egg masses in a communal location, both *L. clamitans* and *L. catesbeianus* produce very large egg masses in large water bodies that are unattached to vegetation, initially floating on the water surface and then sinking prior to hatching, after a day or two (pers. obs.). As a result, we were unable to collect multiple egg masses for either of these species. Instead, *L. clamitans* were collected as pre-foraging hatchlings (Gosner stage 20 [23]) from a pond near Rock Lake (45°31′N, 78°24′W) and a single *L. catesbeianus* egg mass was collected from Lake Sasajewun at the Wildlife Research Station. This *L. catesbeianus* egg mass was placed in a 121 L (84×51×61 cm) Rubbermaid® garbage canister filled approximately half-way with Lake Sasajewun water until hatching. All eggs and hatchlings were transported to Brock University, St. Catharines, ON for experiments.

Hatchlings of all species were housed in 38 L (61×40.6×22.2 cm) Rubbermaid® tubs or 11.4 L (30×25×15 cm) Sterlite® tubs filled with a mixture of filtered pond water and conditioned tap water (carbon-filtered tap water was adjusted to pH 7.0 and aerated for 24 h). As tadpoles grew, they were spread among more tubs so that at testing size there were no more than 20 tadpoles per tub. All tadpoles were maintained on ground Spirulina Algae Discs (Wardley®, Secaucus, New Jersey) and approximately 0.5 L of suspended unicellular green algae (from a lab culture) placed into tubs once a week. Tubs were cleaned of feces every second day and a complete water change performed weekly.

Predators were four *Lepomis gibbosus* sunfish (8–9 cm long) collected in May 2008 from a pond in St. John's Conservation Area, Pelham, Ontario (43°3′N, 79°17′W). When not in the experimental tanks, fish were housed in transparent 10 L (30.5×22.9×17.8 cm) aquaria (Tom Pla-House Clear Vue) filled with approximately 8.5 L of conditioned tap water and were fitted with a sponge filter (Dirt Magnet® Aquarium Filter, Junior Model). To minimize fish handling and stress, fish were kept in outer experimental tanks (see *Experimental Set-up* below) throughout the trial period of each species. When trials finished for the day an air stone and pvc tubing for cover were added to these outer experimental tanks.

Parasites were obtained by collecting *Planorbella trivolvis* snails from a lake at the Glenridge Naturalization Site in Niagara region, Ontario, Canada (43°7′N, 79°14′W). Snails were returned to the lab, housed communally in a small aquarium and fed lettuce *ad libitum*. Cercariae were collected by placing snails in 100 mL disposable Petri dishes filled with conditioned tap water and placed approximately 20 cm away from a 100 W incandescent light bulb. *Echinostoma trivolvis* cercariae were identified by the anterior collar of spines, distinct swimming, and size (for a more detailed description on how to identify cercariae refer to [15], [24]). Two other sympatric parasite species (*E. revoltum* and *Echinoparyphium* sp.) look and behave very similarly to *E. trivolvis,* including the way in which they infect tadpoles [15], [25]. Since we cannot rule out the possibility that some of these were mixed in with *E. trivolvis* cercariae we collected, we hereafter refer to the cercariae more generally as echinostome cercariae. Snails were repeatedly placed in clean water to obtain cercariae, so that all cercariae were used within an hour of leaving the snail host. All animals were kept at room temperature (23–25°C) and on a 14∶10 light∶dark cycle.

### Experimental Set-up

To test the effects of predators and parasites, we used a three-way completely crossed factorial design (predator presence, parasite presence, species) with 20 replicates per treatment combination. For each of the three species tested, treatments were assigned at random to individual tadpoles. Because of phenological differences in anuran species, species could not be randomized and were tested sequentially according to hatching and development timing. Within a species, treatment order was randomized for each replicate. *Lithobates sylvaticus* and *L. clamitans* were used when they reached Gosner stage 26 [23]; fast-developing *L. sylvaticus* were used up to Gosner stage 28. More slowly developing *L. catesbeianus* larvae were used from late Gosner stage 25 to Gosner stage 26. At these early stages, tadpoles of the three species are very similar in size (unpublished data).

Experimental tanks were transparent 10 L aquaria (30.5×22.9×17.8 cm; Tom Pla-House Clear Vue) fitted with an inner 1 L transparent cylindrical container (13 cm in height with a diameter of 13 cm) mounted on top of an 8×5 cm ABS bushing. The inner cylinder housed tadpoles, where they had visual access to fish, but fish could not prey on them. For predator treatments, one fish was placed into the outer tank. Two copy stands, each with a Canon (HV30) camcorder fitted with a polarizing filter and two light sources attached allowed filming of tadpole behavior from above.

All trials were completed each day between 0800 and 1800 hours. To begin a trial, the experimental tank was placed on a copy stand and a tadpole in 150 mL of conditioned water was placed into the inner compartment. A piece of opaque duct piping (24 cm in height with a diameter of 15 cm) fit completely around the inner compartment to block visual detection of external cues in the outer aquarium during the first segment of the trial. The trial began after a five minute acclimatization period, and lasted 35 minutes, divided into three segments. The first 15 minutes of the trial, the tadpole was filmed devoid of any cues/treatment to get an estimate of the individual's baseline activity. This allowed individual variation in activity to be statistically removed from the analysis. At the end of the first segment, 150 mL of one of the four treatments was added to the inner tadpole container (bringing the total volume within the container to 300 mL): (1) control (conditioned water was added), (2) parasite only (150 mL of water inoculated with 36 echinostome cercariae was added to give a treatment density of 36 cercariae per 300 mL), (3) fish only (150 mL of water from sunfish's home tank was added), (4) ‘fish+parasite’ (150 mL of water from the sunfish's home tank was inoculated with 36 cercariae and then added). This density of cercariae used was selected to reflect a moderate level of infection, and is within the range of infection intensities observed within wild caught animals, e.g. [21]. We used only simple predator kairomones (e.g. the “scent” of the predator alone) known to elicit antipredator behaviors in tadpoles [6]; note that diet cues were unattainable for the current study as experienced sunfish refuse to consume unpalatable bullfrog tadpoles [22].

As the treatment water was added, the opaque duct piping was removed exposing the tadpoles to all external visual cues. The second segment of the trial was a 5 minute (while filming) acclimatization period. In the third and final segment of the trial, tadpole post-treatment behavior was recorded for 15 minutes. At the end of the trial, the tadpole was removed to a separate container. The inner chamber was then emptied and wiped clean using 70% ethanol followed by a thorough wipe using dechlorinated water to remove any potential remaining cues prior to the next trial.

### Behavioral Data

All videos were watched and scored by one individual (DS) while blind to treatment. Triplicate estimation of a random subset of videos gave a reliability estimate of ±2% for total time active (n = 5). Total time active in both the 15 minute baseline and 15 minute post-treatment period was quantified using the free software JWatcher 0.9 [26]. Activity was defined as any movement of the tadpole through the water.

Three additional behaviors identified in preliminary trials as associated with cercariae presence were also quantified: (1) Extreme Swimming (number and duration of each bout quantified): tadpole initiated swimming with a fast start and high angular acceleration from a resting state. This behavior was of brief duration (typically 10–20 seconds) and led to little displacement in space. This differs from the burst swimming observed in response to a stimulus, which tends to be linear and leads to a large displacement away from the stimulus (pers. obs.) (2) Body Twisting (number quantified): the tadpole turned its entire body sharply in any direction, bending at the body-tail junction, immediately twisted its body around the dorsal axis and then rolled its body 180° around either its longitudinal or lateral axis. This was a very fast movement taking only a few milliseconds to complete. (3) Tail Flicking (number quantified): tadpole was in a resting state with its tail fully extended, then abruptly bent its tail approximately halfway along its length, and quickly swept the distal portion of the tail forward to one side of the body, and then extended it again. A single tail flick took less than one second to complete. These three behaviors are similar to those observed in other studies that exposed tadpoles to echinostome cercariae (e.g. fast swimming and extremely rapid twisting, turning and tumbling [18], [27]). Total time active was recorded continuously even while these specific behaviors occurred.

### Parasite Load Determination

Tadpoles exposed to parasites in trials were placed individually into 745 mL plastic containers filled with 300 mL of conditioned water for 24 hours. This ensured sufficient time for any cercariae that had attached during the treatment period to encyst within the tissues of the nephric system [14]. Tadpoles were then euthanized with an overdose of the anesthetic MS-222 and preserved in 10% neutral buffered formalin for subsequent dissections. The dissection procedure followed that outlined by Thiemann and Wassersug [28]; all dissections were performed by DS, who was blind to treatment during dissections. Six places within the developing nephric system (the right and left pronephroi, right and left Wolffian ducts, and right and left mesonephroi) were examined and metacercarial cysts were counted.

All methods presented were approved by the Brock University Research Committee on Animal Care Use (AUPP 08-01-01) and collection permits were obtained from the Ministry of Natural Resources in Vineland and Algonquin Park.

### Statistical Analysis

Data were analyzed using R version 2.15 [29]. Since both species and individuals differ in their baseline activity levels [6], [30], we used tadpole activity pre-treatment as a covariate in an ANCOVA that considered the effects of fish (present or absent), parasite (present or absent), species (*L. sylvaticus*, *L. clamitans,* and *L. catesbeianus*), and all interactions on tadpole activity. The response variable was time spent active after the appropriate treatment had been applied. Both pre-treatment activity and post-treatment activity were transformed using (x+0.01)^0.6^; this transformation was arrived at using the powerTransform of the “car” package in R [31] and was done to remove heteroscedasticity that was otherwise present in model residuals.

For measures of extreme swimming, body twisting, and tail flicking, the average number of occurrences for each behavior could not distinguish whether it was a global response expressed by numerous tadpoles to the treatment or one individual performing the behavior numerous times. Therefore, the number of individual tadpoles that performed each behavior at least once (instead of the mean number of occurrences) was analyzed, as we considered this a better metric for assessing whether a particular behavior was related to treatment type. Data were coded so that individuals performing the behavior were given a value = 1 and those that did not were given a value = 0. The likelihood of tadpoles to engage in each behavior in the treatment period was compared using a generalized linear model and binomial distribution (glm in package ‘stats’ of R; [29]). Analysis of deviance was used to assess the significance of higher-order terms, which were eliminated sequentially if they did not contribute significantly to the model fit [32].

To test differences in the mean number of metacercariae that encysted in the nephric systems of each species in the parasite and ‘fish+parasite’ treatments, a negative binomial generalized linear regression was used (glm.nb in package ‘MASS’ of R; [33]).

## Results

### Behavioral Data

The 4-way interaction term between pre-treatment activity, species, fish presence, and parasite presence did not contribute to the model (AIC for the full model: 411.9; AIC for the reduced model: 410.0) and thus this interaction was dropped from the model. However, the model including the 3-way interactions with pre-treatment activity explained significantly more variance than the model omitting these 3-way interactions (ANOVA comparing models, *F_1, 6_* = 2.985, *P* = 0.0098; AIC of model with 3-way interactions removed = 416.42) and we therefore kept these terms in the model (even though the terms themselves did not show significant effects; Table 1).

**Table 1:** Analysis of variance table for final model estimating post-treatment time active in tadpoles in response to effect of species, fish presence or absence, parasite presence or absence and the covariate of time active pre-treatment (actPRE).

	df	SS	MS	F	P
actPRE	1	1.826	1.826	6.2174	0.0134
Species	2	14.438	7.219	24.5736	<0.001
Fish	1	52.985	52.985	180.3670	<0.001
parasite	1	31.696	31.696	107.8972	<0.001
actPRE:species	2	7.101	3.550	12.0857	<0.001
actPRE:fish	1	28.541	28.541	97.1571	<0.001
species:fish	2	27.527	13.763	46.8518	<0.001
actPRE:parasite	1	4.977	4.977	16.9435	<0.001
species:parasite	2	2.367	1.184	4.0294	0.019
fish:parasite	1	2.112	2.112	7.1888	0.008
actPRE:species:fish	2	0.887	0.443	1.5094	0.223
actPRE:species:parasite	2	1.204	0.602	2.0490	0.131
actPRE:fish:parasite	1	0.259	0.259	0.8809	0.349
species:fish:parasite	2	4.290	2.145	7.3022	0.001
Residuals	218	64.040	0.294		

**Figure 2:**
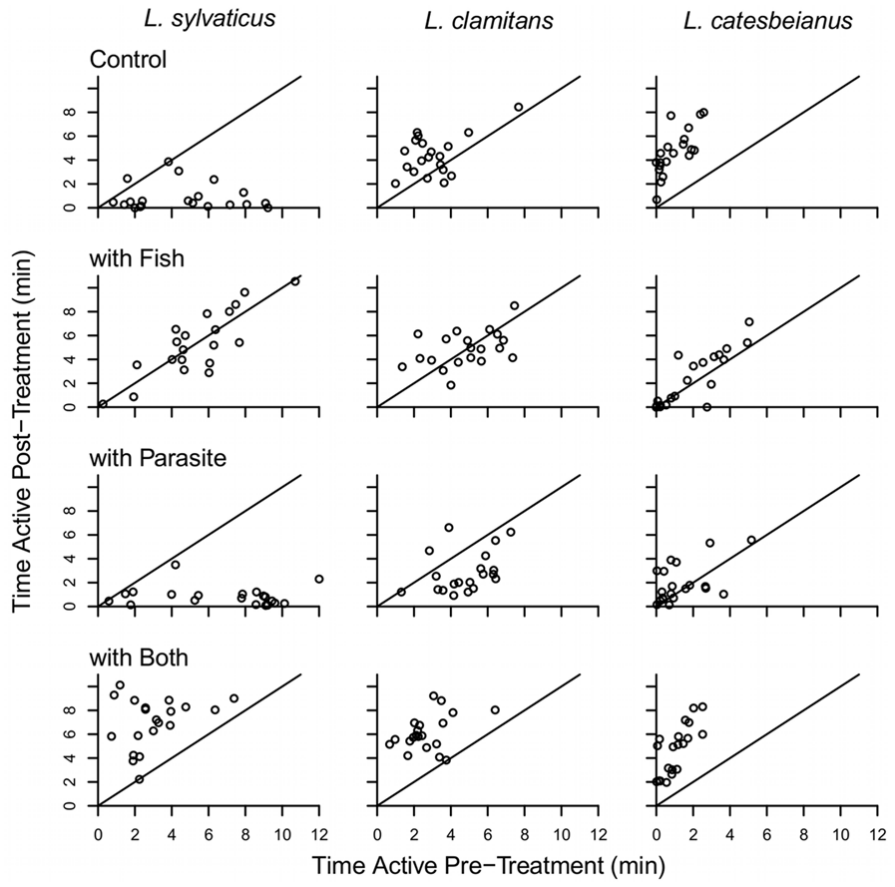
The response of tadpoles for each of the three species to four different treatments. Each of the 12 scatterplots has time active pre-treatment on the x-axis and time active post-treatment on the y-axis. The solid line in each plot indicates where points would fall if post-treatment activity equals pre-treatment activity; points falling left or above this line indicate an increase in activity once the treatment was applied and points falling below or to the right of this line indicate a decrease in activity once the treatment was applied. A significant 3-way interaction between species, fish, and parasite treatments is present.

While activity pre- and post-treatment was correlated in the controls, the correlation was lower in *L. clamitans* (*r* = 0.432) relative to *L. sylvaticus* (*r* = 0.826) and *L. catesbeianus* (*r* = 0.851) (Fig. 2). There was a significant 3-way interaction among species, fish presence, and parasite presence on post-treatment activity (*F_2, 218_* = 7.302, *P*<0.001; Table 1). This arises because while *L. sylvaticus* responds to fish (alone or with parasites) strongly reducing time active, *L. catesbeianus* does not respond to the presence of fish, but does respond strongly, with increased activity, to the presence of parasites (alone or with fish; Fig. 2). All species increased activity in the presence of parasites (Fig. 2). In general, *L. clamitans* post-treatment activity was less affected by the treatments imposed (Fig. 2).

**Figure 3:**
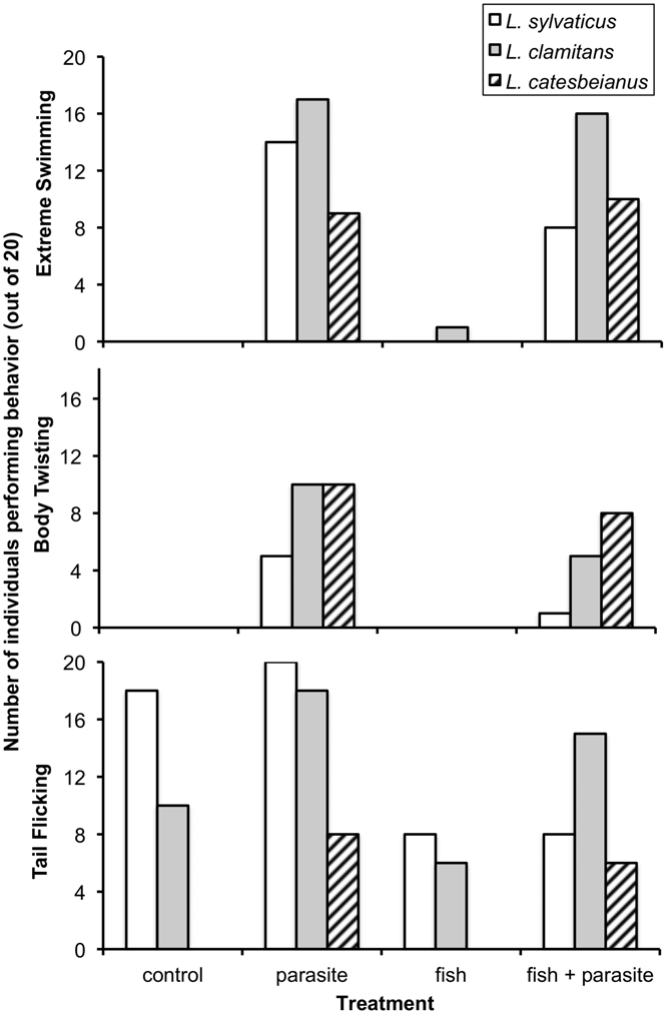
Additional behaviors performed by tadpoles of the three species to four different treatments. Number of tadpoles engaged in a) extreme swimming, b) body twisting, and c) tail flicking behavior post-treatment. See text for behavior descriptions. N = 20 for each treatment combination and data were treated as binomial (i.e. individual in replicate did (score = 1) or did not (score = 0) perform behavior). For both extreme swimming and body twisting, species differed significantly and there was a significant effect of parasite. In body twists, the effect of fish was also significant. For tail flicking, significant species by parasite and species by fish interactions occurred. All effects are based on generalized linear analysis (see text for details).

Of the 360 trials, extreme swimming behavior prior to addition of the treatment (baseline measure) was observed only twice, each time by an *L. sylvaticus* tadpole. Post-treatment, species differed in extreme swimming behavior, with *L. clamitans* performing more extreme swims than the other two species (GLM, reduction in deviance with species effect = 7.209, *P* = 0.027; Fig. 3a). The addition of parasites led to an increased number of tadpoles engaged in extreme swimming for all species (GLM, reduction in deviance when parasite effect added = 133.32, *P*<0.0001; Fig. 3a).

Body twisting occurred only twice during baseline observations (one *L. clamitans* tadpole and one *L. sylvaticus* tadpole, in control and parasite treatments, respectively). Post-treatment, 179 total body twists in 39 individuals were observed. Body twists differed significantly among species (GLM, deviance reduction with species term = 7.88, *P* = 0.019). A strong effect of parasite presence occurred (GLM, deviance reduction with parasite term = 63.347, *P*<0.001) as well as an effect of fish presence (GLM, reduction in deviance with fish treatment = 5.025, *P* = 0.025; Fig. 3b).

All three species in all four treatments engaged in tail flicks during baseline observations, but equally across treatment combinations within each species. Pre-treatment, tail flicking was most likely to occur in *L. sylvaticus* (number of individuals ranged from 13 to 16 in four treatment combinations), was intermediate in *L. clamitans* (3 to 7 individuals) and least likely in *L. catesbeianus* (0 to 2 individuals). After the addition of the treatments, the number of tadpoles performing tail flicks had a significant species by parasite presence interaction (GLM, deviance reduction with addition of species:parasite effect = 15.86, *P* = 0.0004) and a significant species by fish presence interaction (GLM, deviance reduction with addition of species:fish effect = 11.256, *P* = 0.0036). These significant interactions occur because *L. catesbeianus* only performed tail flicking behavior in the presence of parasites, while *L. sylvaticus* decreased tail flicking in the presence of fish (regardless of parasite presence) (Fig. 3c).

### Parasite Success

**Figure 4:**
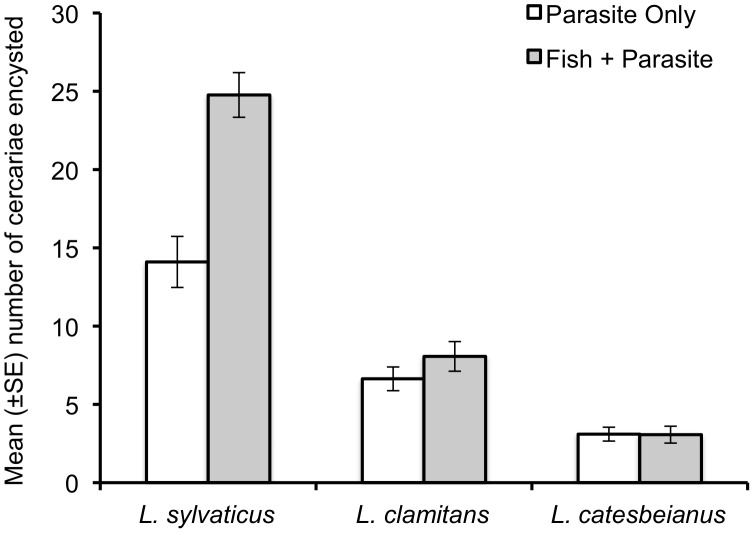
Mean (± SE) number of encysted metacercariae (out of a potential 36 cercariae) for all three species in the parasite only and ‘fish+parasite’ treatments. The species by fish presence interaction was significant (negative binomial GLM analysis of deviance, species by fish; deviance = 206.90, *P* = 0.047).

All *L. sylvaticus* exposed to parasites were infected, while two *L. clamitans* and eight *L. catesbeianus* tadpoles were uninfected. Mean number of metacercariae found in the nephric system of individuals exposed to parasites revealed a significant species by fish presence interaction (GLM, deviance reduction with addition of species:fish term = 6.135, *P* = 0.047; Fig. 4). Overall, *L. sylvaticus* had more encysted metacercariae than each of the other species, and significantly more cercariae successfully encysted in those *L. sylvaticus* tadpoles exposed to the ‘fish+parasite’ treatment compared to the parasite only treatment (Fig. 4). *Lithobates clamitans* larvae also had more encysted metacercariae than *L. catesbeianus*, but the difference between fish present and fish absent treatments did not differ significantly for either *L. clamitans or L. catesbeianus* (based on model contrasts; Fig. 4).

Control tadpoles were not dissected to check for potential parasite infection, however, in multiple subsequent parasitism experiments using the same amphibian and parasite species (unrelated to the current study), we have never found parasites in the control tadpoles (pers. obs.)

## Discussion

Our behavioral results indicate that larvae of three *Lithobates* species respond differently to the trade-off imposed by the simultaneous presence of both a predator and parasites. An anti-predator response dominates behavior of *L. sylvaticus* in the presence of both fish and parasites, and anti-parasite behavior dominates the response of *L. catesbeianus* in the presence of both fish and parasites. The behavior of *L. clamitans* in the combined fish and parasite treatment was also biased toward anti-parasite behavior, but to a lesser extent than in *L. catesbeianus*.

Larvae of all species increased time active in the presence of parasites alone, clearly demonstrating that tadpoles respond to the presence of parasites with increased activity. Further, this increased activity is effective in reducing parasitism, as indicated by the significant difference in encysted echinostomes between the parasite only versus the ‘fish+parasite’ treatment for *L. sylvaticus* (the species with the most dramatic change in time active between the parasite only and the fish+parasite treatments). Koprivnikar et al. [16] also observed a relationship between activity and parasitism in *L. clamitans*, with tadpoles more likely to be infected when they were less active (the number of cysts per tadpole did not differ, but tadpoles were exposed to only five cercariae). Similarly, *Pseudacris regilla* anesthetized to prevent behavioral response have a greater infection prevalence and intensity from *Echinostoma* than conspecifics not anesthetized [19]. Activity level cannot fully explain differences in parasite intensity in our study: all species were equally active in the parasite only treatment, yet *L. sylvaticus* had greater numbers of encysted parasites than either *L. clamitans* or *L. catesbeianus* (Fig. 4). Fewer *L. sylvaticus* tadpoles displayed body twist behavior than either *L. clamitans* or *L. catesbeianus*. Thus, one possible explanation for difference in parasite intensity among species is that body twists are an especially good behavioral mechanism for dislodging echinostome cercariae. Unfortunately, the methodology of the current study did not allow us to correlate an individual's behavior with its subsequent parasite numbers. Future studies doing this will be useful in clarifying the role of these behaviors in avoiding/dislodging potential parasites. That said, it seems unlikely that this one behavioral difference is sufficient to explain the increased number of successful parasites in *L. sylvaticus* and it does not explain observed differences in parasite intensity between *L. clamitans* and *L. catesbeianus*.

As with differences in predators among habitat types, it is possible that the three species tested in this study experience different levels of parasite exposure in the wild. While trematode parasites are common and prevalent in amphibian populations [16], [34], temporary pond breeders such as *L. sylvaticus* may encounter cercariae of echinostomes less frequently or for a shorter period of time than either *L. clamitans* or *L. catesbeianus* (although *L. sylvaticus* have been observed with *Echinostoma* cysts in the field [35]). For example, *L. sylvaticus* metamorphose within a single season where both *L. clamitans* and *L. catesbeianus* typically overwinter as tadpoles [36], spending an additional summer in the pond, which in turns exposes them to trematode parasites for a much longer period than *L. sylvaticus* tadpoles. In addition, the first intermediate host of *E. trivolvis* is the snail *P. trivolvis* and this species is most often found in permanent pond settings, namely well vegetated lentic or still waters and farm ponds, dams, lakes [37]. As *L. sylvaticus* often inhabit temporary ponds that can dry by the end of the season, these ponds are generally not suitable habitats for *P. trivolvis* snails. However, there are ponds that are more permanent in that they do not dry regularly, yet lack major vertebrate predators such as fish; these ponds would be suitable habitats for both *P. trivolvis* and breeding *L. sylvaticus* (in fact, our *L. sylvaticus* eggs were collected from a large permanent lake that lacks fish predators). In ponds that dry often, other species of snails (e.g. *Pseudosuccinea columella*) that can undergo aestivation during dry periods, persist along with other macroparasites that also use larval amphibians as a secondary host (e.g. *Telorchis* spp.) [38]. Further studies of parasite distributions among ponds that vary in permanence will allow us to better relate putative antiparasite behaviors to ecologically relevant conditions.

We demonstrate here that the presence of multiple enemies (parasites and predators) in an environment lead to non-additive behavioral responses in tadpoles that are species-specific. An important caveat to this interpretation of species-specific effects is that we had limited genetic diversity of *L. catesbeianus* and *L. clamitans* tadpoles (our *L. catesbeianus* individuals were from a single egg mass). While this is far from ideal, the egg mass was collected at random (through extensive blind dip-netting in a large lake) and we have no reason to believe that these individuals were not representative of the species response. Nonetheless, within species variation in response to predators and parasites is known to occur, at least in *L. sylvaticus* [30]. Thus future studies replicating the observed differences among species and expanding the species considered are necessary. In addition, future studies should also include other potentially important variables such as differences in temperature preference among species, differences in oxygen concentrations within the different habitats and habitat complexity, as these factors may influences susceptibility to both predators and parasites. Regardless, our data add to a growing body of evidence that suggests parasites and pathogens in animal communities can have trait-mediated indirect effects that influence predator-prey interactions. Contrary behavioral responses to parasites and predators have been observed in damselfly larvae [20] and *Daphnia* [39], as well as tadpoles [27], [40], [41]. While the potential for non-additive and trait-mediated interactive effects among predators that share prey within a community is well-recognized, consideration of parasites within this context has been neglected until recently [10].

## Conclusions

In this study we demonstrate three important and novel results regarding tadpoles, their predators and their parasites: i) in the presence of free-swimming trematode cercariae, tadpoles increase general activity and some specific behaviors such as body twisting; ii) this behavioral response to parasites corresponds to decreased parasite encystment success; iii) species respond differentially to the trade-off imposed by the presence of both parasites and fish predators as predicted based on the presence of alternative (non-behavioral) antipredator traits. Our results add to a growing body of evidence that as well as the potential for interactions among the direct effects of predators and parasites, substantial indirect interactive effects may occur through trait-mediated effects, particularly behavioral responses of potential prey/hosts to the presence of predators and parasites. Further, these effects are likely to be species-specific, dependent on the suite of traits species possess that affect predation and parasitism risk. Recognition of the increased complexity of interactions among species within a community places greater urgency on our ability to quantify and understand such interactions if we are to predict the effects of the changes in biodiversity currently affecting communities at unprecedented rates [42].
