# Correction: The Behavioral Response of Larval Amphibians (Ranidae) to Threats from Predators and Parasites

**DOI:** 10.1371/journal.pone.0203252

**Published:** 2018-08-27

**Authors:** Dorina Szuroczki, Jean M. L. Richardson

In Fig 2, the treatment labels were erroneously swapped. Please see the corrected Fig 2 here.

**Fig 2:**
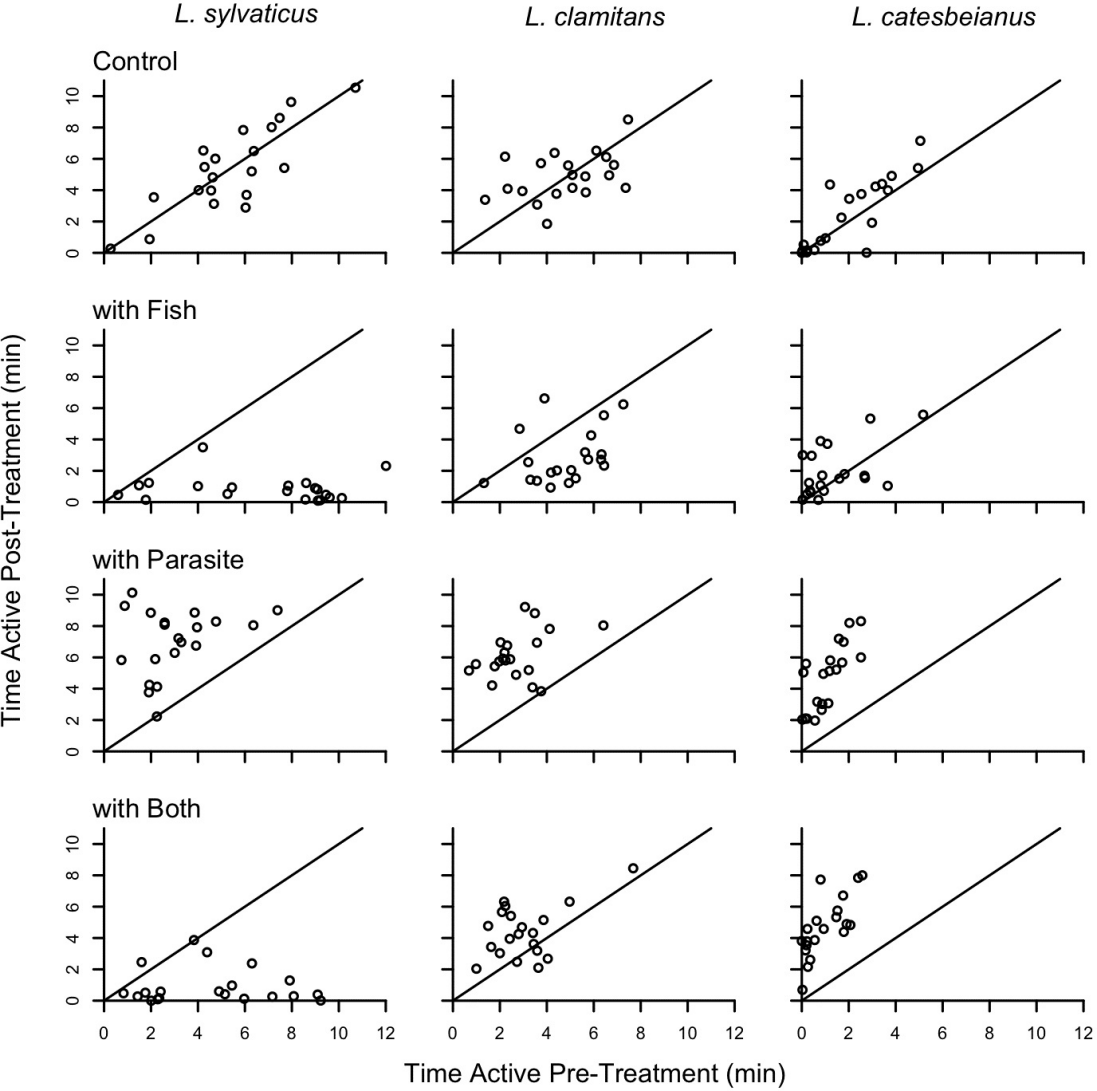
The response of tadpoles for each of the three species to four different treatments. Each of the 12 scatterplots has time active pre-treatment on the x-axis and time active post-treatment on the y-axis. The solid line in each plot indicates where points would fall if post-treatment activity equals pre-treatment activity; points falling left or above this line indicate an increase in activity once the treatment was applied and points falling below or to the right of this line indicate a decrease in activity once the treatment was applied. A significant 3-way interaction between species, fish, and parasite treatments is present.
